# Non-invasive Beta-cell Imaging: Visualization, Quantification, and Beyond

**DOI:** 10.3389/fendo.2021.714348

**Published:** 2021-06-25

**Authors:** Takaaki Murakami, Hiroyuki Fujimoto, Nobuya Inagaki

**Affiliations:** ^1^ Department of Diabetes, Endocrinology and Nutrition, Kyoto University Graduate School of Medicine, Kyoto, Japan; ^2^ Radioisotope Research Center, Agency of Health, Safety and Environment, Kyoto University, Kyoto, Japan

**Keywords:** beta-cell imaging, glucagon-like peptide-1, exendin, positron emission tomography, single photon emission computed tomography, β-cell mass, islet transplantation, diabetes mellitus

## Abstract

Pancreatic beta (β)-cell dysfunction and reduced mass play a central role in the development and progression of diabetes mellitus. Conventional histological β-cell mass (BCM) analysis is invasive and limited to cross-sectional observations in a restricted sampling area. However, the non-invasive evaluation of BCM remains elusive, and practical *in vivo* and clinical techniques for β-cell-specific imaging are yet to be established. The lack of such techniques hampers a deeper understanding of the pathophysiological role of BCM in diabetes, the implementation of personalized BCM-based diabetes management, and the development of antidiabetic therapies targeting BCM preservation and restoration. Nuclear medical techniques have recently triggered a major leap in this field. In particular, radioisotope-labeled probes using exendin peptides that include glucagon-like peptide-1 receptor (GLP-1R) agonist and antagonist have been employed in positron emission tomography and single-photon emission computed tomography. These probes have demonstrated high specificity to β cells and provide clear images accurately showing uptake in the pancreas and transplanted islets in preclinical *in vivo* and clinical studies. One of these probes, ^111^indium-labeled exendin-4 derivative ([Lys^12^(^111^In-BnDTPA-Ahx)]exendin-4), has captured the longitudinal changes in BCM during the development and progression of diabetes and under antidiabetic therapies in various mouse models of type 1 and type 2 diabetes mellitus. GLP-1R-targeted imaging is therefore a promising tool for non-invasive BCM evaluation. This review focuses on recent advances in non-invasive *in vivo* β-cell imaging for BCM evaluation in the field of diabetes; in particular, the exendin-based GLP-1R-targeted nuclear medicine techniques.

## Introduction

The number of patients with diabetes worldwide is on the rise. The estimated prevalence of patients with diabetes 20 years of age or older has risen from 151 million to 463 million from 2000 to 2019, and it is predicted to increase to 700 million by 2045 (1). To control this increase, we need a detailed understanding of the pathogenesis of diabetes and to develop new diagnostic strategies and policies for treating the disease.

The reduction of pancreatic beta (β)-cell mass (BCM) and β-cell function involves the onset and progression of diabetes; BCM plays a central role in the pathophysiology of both type 1 and type 2 diabetes mellitus (2–4). In type 1 diabetes mellitus, the time-course patterns of β-cell destruction vary from the prominent type to latent autoimmune diabetes in adults or slowly progressive insulin-dependent diabetes mellitus (5–7). Residual BCM is therefore an important factor in managing type 1 diabetes mellitus, whereas viable graft islet volume largely affects the outcomes in islet cell transplantation (8). In type 2 diabetes mellitus, substantially reduced BCM is observed, even at diagnosis, and can affect the responsiveness of antidiabetic therapy (3, 9–11). Therefore, a BCM evaluation leads to not only a deeper and more concise understanding of the individual’s diabetic state but also the development and evaluation of antidiabetic therapy aimed at BCM restoration and protection. Although conventional methods for evaluating *in vivo* BCM are difficult for practical use due to their invasiveness and unsatisfactory β-cell specificity, non-invasive pancreatic β-cell imaging using nuclear medicine techniques have emerged in recent years. In particular, radioisotope-labeled probes using exendin peptides [including glucagon-like peptide-1 (GLP-1) receptor agonist and antagonist] have been promising tools for positron emission tomography (PET) and single-photon emission computed tomography (SPECT) imaging. A number of these probes, including ^111^indium-labeled exendin-4 derivative {[Lys^12^(^111^In-BnDTPA-Ahx)]exendin-4}, have demonstrated the visualization of the mouse pancreas and its image analysis for BCM quantification and thereby have shown potential for clinical use (12–15). This review focuses on the recent advances in non-invasive *in vivo* β-cell imaging and BCM evaluation for type 1 and type 2 diabetes mellitus, especially exendin-based GLP-1R-targeted nuclear medicine techniques.

## The Urgent Need for Non-Invasive β-Cell Mass Evaluation in Diabetes

**Figure 1:**
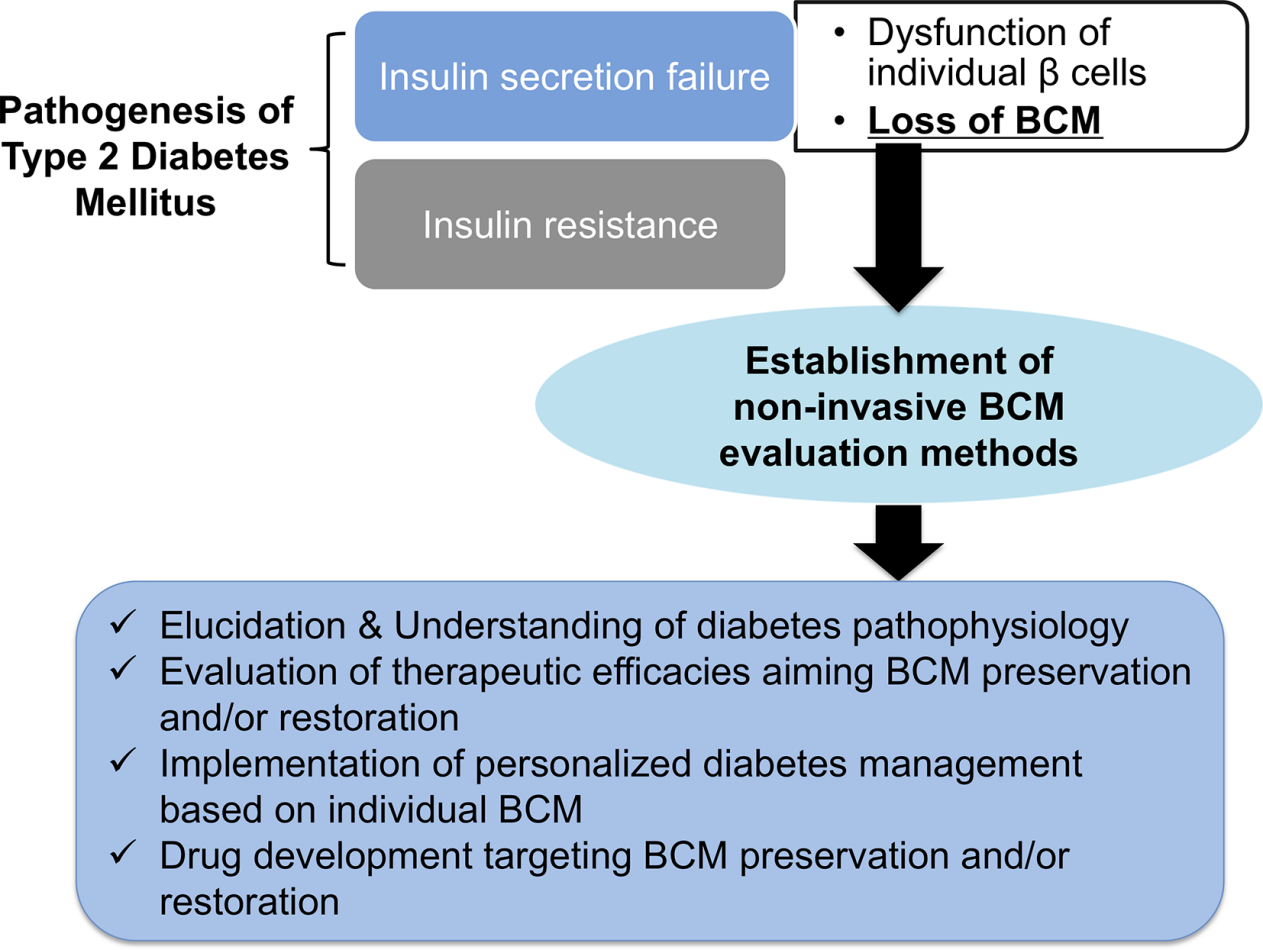
The expected benefits of non-invasive β-cell mass (BCM) evaluation. Loss of BCM has a central role in the onset and progression of type 2 diabetes mellitus. The establishment of non-invasive BCM evaluation methods will open the door to elucidating and understanding the pathophysiology of diabetes, the evaluation of therapeutic efficacies aimed at preserving and restoring BCM, the implementation of personalized diabetes management based on individual BCM, and drug development targeting BCM preservation and restoration.

Both type 1 and type 2 diabetes mellitus are characterized by a progressive reduction in BCM (16). Although the evaluation of pancreatic β-cell function is essential for understanding insulin secretion failure, the systemic β-cell function consists not only of the ability of individual β cells to secrete insulin but also the regulation of BCM (**Figure 1**). However, conventional markers such as plasma and urine C-peptide levels do not provide direct information regarding BCM and do not discriminate changes in BCM from those of individual β-cell function. Therefore, the exact relationship between BCM and β-cell function in the onset and progression of diabetes remains unclear (17–19). Direct information regarding BCM itself provides a novel parameter for understanding the pathophysiology of diabetes and evaluating the efficacy of new and existing antidiabetic therapy. However, the conventional histological method for evaluating BCM using pancreas samples through autopsy or surgery is invasive and is limited to cross-sectional observations (20). This method also raises certain concerns in terms of the restricted sampling area and staining evenness in view of representing the BCM of the entire pancreas (20). A novel technique for non-invasive BCM monitoring is therefore an urgent need in basic research and clinical fields.

In type 1 diabetes mellitus, pancreatic β cells are predominantly destroyed by autoimmune attacks, which result in markedly reduced BCM and severe insulin deficiency. However, the remaining viable β cells, including non-functional ones, are observed in over 30% of patients with long-duration type 1 diabetes mellitus (18, 21, 22). Moreover, slices of pancreatic tissue from organ donors with type 1 diabetes mellitus revealed that BCM loss might be preceded by a decline in β-cell function and have a varying contribution to the pathogenesis of type 1 diabetes mellitus (23). Recent studies have revealed that certain patients present with slowly progressive types of type 1 diabetes mellitus, such as latent autoimmune diabetes in adults and slowly progressive insulin-dependent diabetes mellitus, in which systemic β-cell function is persistently preserved, although the natural history of BCM changes in these types remains to be investigated (5–7). These findings suggest the range of remaining BCM in individuals with type 1 diabetes mellitus and increase the clinical importance of evaluating BCM. In addition, BCM can be a potential target for preservation and restoration in future therapeutic interventions for type 1 diabetes mellitus. A BCM evaluation is therefore essential for broadening the insights into its pathophysiology and developing a new therapeutic strategy for type 1 diabetes mellitus.

Islet transplantation, in which isolated donor islets are infused into the portal vein, has become a promising treatment option for patients with type 1 diabetes mellitus (24, 25). Although islet transplantation can improve glycemic control and even achieve insulin independence (25, 26), a sufficient volume of islet grafts is required to achieve insulin independence. The post-transplantation loss of islet grafts is often observed, which might require repeated islet transplantations (8, 26, 27). A non-invasive imaging method is therefore needed to monitor these islets over time. Stem-cell-derived transplantation, encapsulation devices, and subcutaneous implantation have received significant attention recently as approaches for solving the problem of lack of donors and to minimalize invasiveness (26, 28–30). Non-invasive β-cell imaging is useful for evaluating and improving the efficacy of these techniques.

In type 2 diabetes mellitus, a hypothetic model of BCM changes has been proposed; type 2 diabetes mellitus occurs in response to a reduction in BCM following a temporary compensatory increase in BCM induced by obesity and insulin resistance. BCM subsequently decreases progressively (2, 3). This hypothetic model is, however, simply a patchwork model based on the implications of cross-sectional studies, given that the method for evaluating BCM has been limited to a pathological type, using surgically resected or postmortem pancreatic samples (20). Butler AE et al. investigated BCM using post-mortem pancreatic sections and reported that BCM in type 2 diabetes mellitus was reduced by 63% in cases of obesity and by 41% in cases without obesity compared with those cases with normal glucose tolerance (10). Interestingly, BCM in impaired fasting glucose conditions also showed an approximately 40% reduction compared with that in healthy participants (10). In other autopsy studies of Asian populations such as Japan and South Korea, a 30–50% reduction in BCM was observed in patients with type 2 diabetes mellitus compared with those with normal glucose tolerance (31, 32). These reports suggest that the onset and progression of type 2 diabetes mellitus is involved not only in the decline in β-cell function but also in the reduction in BCM (33, 34). In this context, preserving or restoring BCM is a critical strategy for the prevention and long-term management of the disease (3, 9). Moreover, individual BCM information can provide a chance to optimize antidiabetic therapy individually, which can open the door to truly personalized precision medicine in the field of diabetes. The elucidation of the individual’s remaining BCM and the effects of antidiabetic agents on BCM is of significant value (20, 35). These studies also raise the clinical issue that a non-negligible reduction in BCM might have already occurred by the time type 2 diabetes mellitus is clinically diagnosed (10, 20). To address this unmet clinical need, an earlier recognition and monitoring of BCM changes are essential for preventing and preemptively managing type 2 diabetes mellitus. Urgent needs for non-invasive BCM evaluation should therefore be noted (**Figure 1**).

## Challenges of Non-Invasive β-Cell-Specific Imaging

Despite the need for and the potential of non-invasive β-cell imaging and quantification, the feasibility of such technologies has been hampered by numerous obstacles, one of which is the small and scattered area comprising β cells in the total pancreas. Pancreatic β cells constitute only 1–3% of the total pancreatic mass. β cells also constitute the islets of Langerhans, which are heterogeneously distributed throughout the pancreas and are composed of various other cell types such as alpha, delta, and pancreatic polypeptide cells (34, 36). Another major obstacle is the spatial resolution of clinical imaging modalities, given that the islets of Langerhans are approximately 40–300 μm in diameter. *In vivo* β-cell visualization and quantification require imaging modalities with sufficiently high spatial resolution to image individual islets, and no widely available clinical modality [including computed tomography (CT) and magnetic resonance imaging (MRI)] has satisfied this requirement (17). Therefore, instead of resolving single islets, the strategy has been to measure total pancreas signals from tracer molecules highly specific to β cells and provide an estimated BCM. In this context, nuclear medicine imaging techniques such as SPECT and PET have attracted attention (20, 37) due to their ability to detect their radioisotope-labeled probes at picomolar ranges, despite their limited spatial resolutions (38). The high specificity to β cells of high-sensitivity imaging modalities combined with radioisotope-labeled probes outweighs the shortcomings of their spatial resolution (17, 39). Accordingly, SPECT and PET have been investigated for the application to *in vivo* β-cell-specific imaging.

## Exploration of Ideal Probe Targets in *In Vivo* β-Cell Imaging

**Figure 2:**
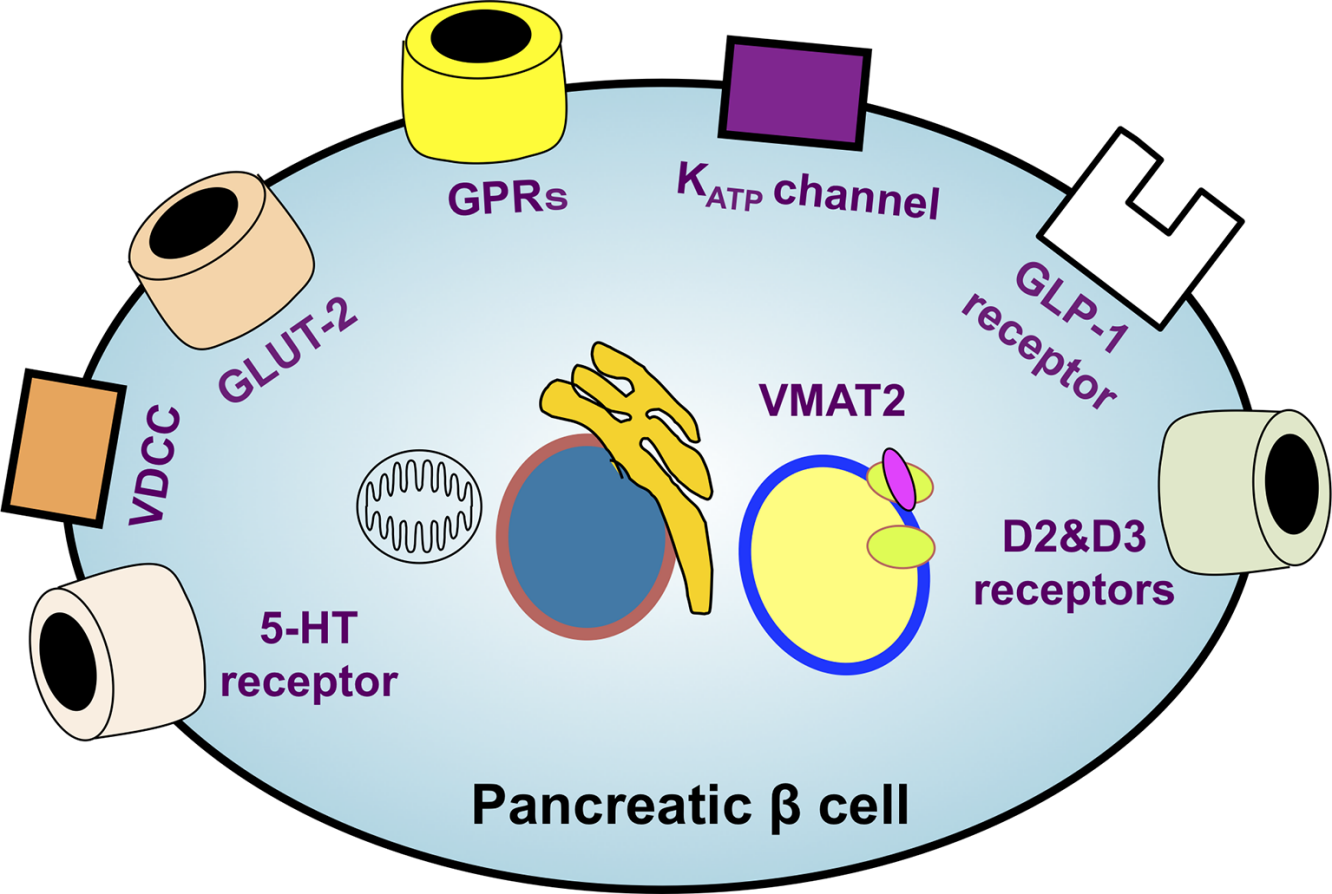
An overview of the potential targets of β-cell-specific imaging probes. The identification of ideal probe targets highly specific to β cells is essential for realizing β-cell imaging. The various molecules whose expression is observed specifically in β cells have been explored. SUR1, glucose transporter 2 (GLUT-2), voltage-dependent calcium channel (VDCC), G protein-coupled receptors (GPRs), D2 and D3 dopamine receptors, serotonergic system, vesicular monoamine transporter 2 (VMAT2), and GLP-1 receptors have been reported as leading potential probe targets. K^ATP^ channel, ATP-sensitive potassium channel; 5-HT receptor, 5-hydroxytryptamine receptor.

The identification of ideal probe targets highly specific to β cells is essential for performing β-cell SPECT and PET imaging. Therefore, the various molecules whose expression is observed specifically in β cells have been explored, and those ligands, substrates, and antibodies have been investigated as putative probes for β-cell imaging. To date, sulfonylurea receptor 1 (SUR1) (40, 41), glucose transporter 2 (42), voltage-dependent calcium channel (43), G protein-coupled receptor 44 (GPR44) (44, 45), D2 and D3 dopamine receptors (46, 47), serotonergic system (48–51), vesicular monoamine transporter 2 (VMAT2) (52, 53), and GLP-1 receptor (GLP-1R) (54, 55) have been reported as potential probe targets (**Figure 2**) (56).

SUR1 is a subunit of ATP-dependent potassium channels and is specifically expressed in β cells except in the brain. Given that sulfonylureas are known for their binding affinities to SUR1 and in light of their clinical use experience, a number of radiolabeled sulfonylurea derivatives such as glibenclamide and glipizide have been investigated. However, these probes have failed to achieve sufficient specificity to β cells; low accumulations in the pancreas and high background signals (57, 58). Although a mitiglinide derivative has been reported as a potential β-cell imaging probe with higher specificity (40), none of the available probes targeting SUR1 are currently feasible.

D2 and D3 dopamine receptors involve glucose-stimulated insulin secretion in β cells (59) and have been suggested as a target for β-cell imaging (46). PET imaging with the receptor agonist 3,4,4a,5,6,10b-hexahydro-2H-naphtho(1,2-b)(1,4)oxazin-9-ol was examined in patients with type 1 diabetes mellitus (46), demonstrating reasonable pancreatic uptake and non-negligible uptake in the spleen, located near the tail of the pancreas. A substantial overlapping of probe accumulations in the pancreas has been observed between patients with type 1 diabetes mellitus and healthy participants (46). Another probe, [^18^F]dihydroxyphenylalanine [(^18^F)DOPA], has been investigated for nesidioblastosis, with several groups reporting the successful imaging of responsible foci. However, [^18^F]DOPA was taken up in both endocrine and exocrine pancreatic cells and showed high background signals, which suggests a limited potential use for this probe (60).

(^11^C)5-hydroxytryptophane [(^11^C)HTP] is a tracer employed for evaluating serotonin biosynthesis and is metabolized by dopamine decarboxylase to (^11^C)serotonin. Given that serotonin is accumulated in β cells, [^11^C]HTP PET has been investigated in β-cell imaging, demonstrating substantially reduced accumulation in the pancreas of patients with type 1 diabetes mellitus (48). (^11^C)HTP PET also demonstrated its potential utility for the longitudinal observation of islet mass in type 1 diabetes mellitus and islet transplantation (49, 51). However, conflicting reports have suggested that HTP is accumulated in other pancreatic endocrine and exocrine cells and have encountered difficulties in distinguishing BCM between healthy participants and patients with diabetes due to the large overlap (17, 61, 62).

VMAT2 is an integral membrane protein for neurotransmitter transport. The derivatives of dihydrotetrabenazine (DTBZ), a ligand of VMAT2, have been actively researched as potential β-cell imaging probes. However, most DTBZ derivatives have demonstrated high exocrine-islet ratios in the pancreas, lacking the specific accumulation in β cells. (^18^F)fluoropropyl-DTBZ has been employed for evaluating BCM in patients with type 1 diabetes, showing lower accumulations in the pancreas compared with those of healthy participants (52). However, non-specific binding was observed not only in the liver and spleen but also in the pancreas, which could significantly affect the accuracy of the BCM evaluation and could overestimate BCM (52). Most signals of the DTBZ-related probes originated from delta and PP cells, which also expressed VMAT2, and this non-specific binding was higher in patients with diabetes (52, 63, 64). These probes should therefore be further optimized for BCM evaluation.

## Visualization of β Cells: Glucagon-Like Peptide-1 Receptor-Targeted Imaging

GLP-1R is a G-protein-coupled receptor that plays a key role in glucose metabolism and is a major therapeutic target for diabetes. GLP-1R is considerably expressed in β cells, whereas its expression level in other endocrine cells has been reported as absent or low (65). GLP-1R expression in pancreatic exocrine cells is low in humans, although certain animal models such as pigs exhibit relatively high GLP-1R expression in the exocrine pancreas (66). GLP-1R has therefore been the most actively investigated viable target of β-cell imaging probes.

**Figure 3:**
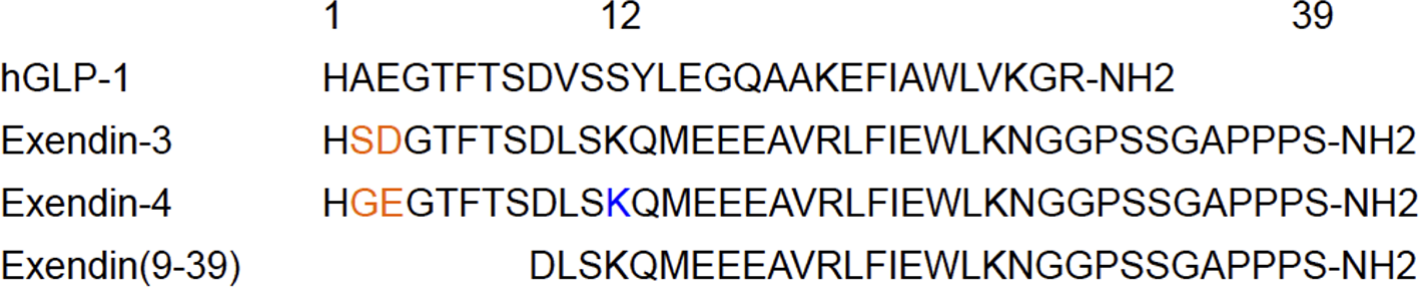
An overview of the leading compounds for GLP-1R-targeted probes. In the development of GLP-1R-targeted imaging probes, exendin-related peptides such as exendin-3, exendin-4, and exendin (9-39) have been investigated as promising compounds. These compounds have approximately 50% homology with human GLP-1 (hGLP-1) and show high stability *in vivo* and high affinity to GLP-1R, which qualifies their potential as tracers for *in vivo* β-cell imaging. Exendin-4 differs from exendin-3 by two amino acid substitutions (in orange). C-terminally modified derivatives tended to show superior specificity, whereas the modified derivatives on the residue Lys^12^ of exendin-4 (in blue) demonstrated high affinity to GLP-1R and *in vivo* stability as *in vivo* imaging probes for β cells.

GLP-1, an endogenous GLP-1R ligand peptide, ameliorates glucose-dependent insulin secretion, whereas having a very short plasma half-life due to the rapid degradation by dipeptidyl peptidase-4 (67). In line with efforts to improve the *in vivo* stability against dipeptidyl peptidase-4, exendin peptides (originally found in the saliva of *Heloderma suspectum*) have been synthesized and have shown high *in vivo* stability with a high affinity to GLP-1R (68). Whereas exendin (9-39) is a GLP-1 antagonist, exendin-3 and exendin-4 are GLP-1R agonists. Exendin-4 differs from exendin-3 by two amino acid substitutions (Gly2-Glu3 in place of Ser2-Asp3) but is otherwise identical (**Figure 3**). Moreover, exendin-4 has been successfully employed in the clinical treatment of type 2 diabetes mellitus, which qualified the potential clinical stability and safety of exendin-based probes for β-cell imaging. Exendin-related peptides, especially exendin-4, have therefore become the lead compounds in the development of GLP-1R-targeted probes for β-cell imaging, and various modified peptides have been investigated as potential imaging probes.

As for *in vivo* β-cell visualization, the pioneer study with exendin-based probe for GLP-1R was reported with radioiodinated exendin-3 (69). Biodistribution studies of [^123^I]exendin-3 in rats harboring rat insulinoma cells (RINm5F) have shown rapid blood clearance and uptake of the radiotracer into the tumor and pancreas, which could be detected by SPECT. ^111^In-labeled exendin-3, {Lys^40^[(^111^In)DTPA]}exendin-3, was subsequently synthesized, given that the high sensitivity of ^111^In-labeled tracers allowed for the administration of small amounts of tracer to prevent receptor saturation and adverse effects in SPECT (70). In a controlled, unilaterally nephrectomized mouse study, autoradiography showed the specific uptake of {Lys^40^[(^111^In)DTPA]}exendin-3 in insulin-expressing cells, with the highest uptake in the pancreas and lungs followed by the kidneys (71). In another study, diabetic rats showed ^111^In-[Lys^40^]exendin-3 uptake in the pancreas (12). ^68^Gallium (Ga)-labeled exendin-3 has also been developed as a probe for PET imaging (72). In most of the designs of exendin-3 probes for SPECT and PET, the residue Lys^40^ of exendin-3 was conjugated with the radioisotope-labeling structures. Although exendin-3 probes showed successful *in vivo* visualization of the pancreas, further research to improve the stability is warranted (39).

Subsequently, exendin (9-39) also has been expected as a potent skeleton of GLP-1R-targeted probe for β-cell imaging, and its derivatives have been investigated. One of the considerable advantages of exendin (9-39) is its ability to avoid hypoglycemia due to GLP-1R activation. A preclinical study of ^125^Iodine-Bolton-Hunter ([^125^I]BH)-labeled exendin (9-39) exhibited good affinity to GLP-1R and high uptake in mice pancreas (54). However, the uptake in pancreatic β cells might be species-dependent because the pancreatic uptake of (^125^I)BH-exendin (9-39) differed in human and mouse tissues (73). Based on the influence of the BH labeling site on the target properties, BH labeling on Lys^19^ showed comparable affinity in human and rat tissues (74). In addition, a moiety of (^111^In) diethylenetriaminepentaacetic acid (DTPA) or tetraazacyclododecane tetraacetic acid (DOTA) *via* aminohexanoic acid linker (Ahx) has been introduced to obtain clearer SPECT images of the pancreas. In accordance with (^125^I)BH reagents, partial modifications of exendin (9–39) at lysine residues and the N-terminus have been considered in the development of [^111^In]DTPA-labeled derivatives (75). However, a crystal structure analysis suggested that modification of the lysine at position 27 would not improve the imaging of GLP-1R-positive tissues (75, 76). ^111^In-[Lys^40^(Ahx-DTPA)NH2]exendin (9–39) probe showed reasonable affinity to GLP-1R *in vitro*, whereas a biodistribution study demonstrated low specific uptake without efficient retention or internalization of the probe, with lower accumulation in rat insulinoma INS-1 tumors and lower INS-1 tumor/pancreas contrast ratio compared with GLP-1R agonist-based counterparts (77). (^111^In)benzyl (Bn)DTPA-exendin (9-39) demonstrated higher affinity for GLP-1R compared with its parent compound, exendin (9-39), and clearly visualized INS-1 tumors in mice (75). Compared with ^111^In-[Lys^40^(Ahx-DTPA)NH2]exendin (9-39), higher levels of tumor accumulation and uptake ratios between tumors and surrounding organs were observed in a biodistribution study involving INS-1 tumor-bearing mice (75). As for PET imaging, fluorobenzoyl (FB)-modified exendin (9-39) derivatives have been synthesized (15, 78). (^18^F)FB40-exendin (9-39) showed moderate affinity to GLP-1R, visualized the mouse pancreas, and exhibited superior potentials to other ^18^F-labeled exendin (9-39) derivatives on different conjugating sites (Lys^12^ and Lys^27^) (15). Further enhancement of pancreatic uptake and specific binding to GLP-1R will lead to a clearer visualization of pancreatic β cells in exendin (9-39)-based PET imaging (15).

**Figure 4:**
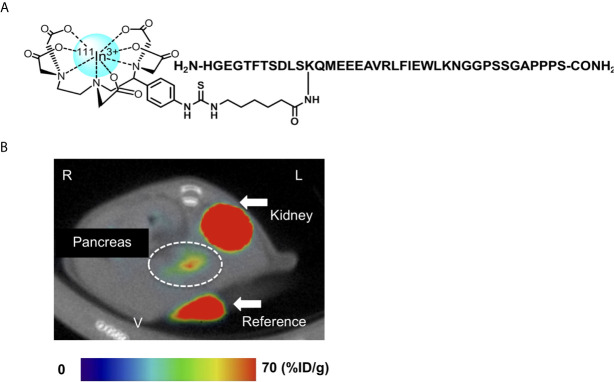
An ^111^Indium(In)-labeled exendin-4 derivative: [Lys^12^(^111^In-BnDTPA-Ahx)]exendin-4. **(A)** Chemical structure of [Lys^12^(^111^In-BnDTPA-Ahx)]exendin-4. Exendin-4 was labeled with ^111^In *via* isothiocyanate-benzyl-diethylenetriaminepentaacetic acid (BnDTPA) and 6-aminohexanoic (Ahx) attached to the epsilon amino group at the lysine-12 residue. **(B)** Representative *in vivo* axial abdominal image of [Lys^12^(^111^In-BnDTPA-Ahx)]exendin-4 single-photon emission computed tomography (SPECT)/computed tomography (CT) in a mouse. [Lys^12^(^111^In-BnDTPA-Ahx)]exendin-4 successfully visualized the pancreas (white dotted circle). Maximum to minimum SPECT intensity: red > orange > yellow > green > blue > black. R, right; L, left; V, ventral.

Exendin-4 has been the most promising compound in the development of GLP-1R-targeted probes for β-cell imaging. Although various modifications of exendin-4 have been investigated, the representative potent exendin-4-based probes are those with the modification of lysine at position 12 and those with the C-terminal modification of adding a lysine at position 40 for SPECT imaging (12, 14). [Lys^40^(Ahx-DTPA-^111^In)NH2]exendin-4 exhibited higher and more stable uptake in the murine pancreas compared with ^111^In-[Lys^40^(Ahx-DTPA)NH2]exendin (9-39) and visualized the pancreas with SPECT in healthy participants and patients with type 1 diabetes mellitus (12, 77). A clinical study employed [Lys^40^(Ahx-DTPA-^111^In)NH2]exendin-4 to detect insulinoma and showed successful visualization and clinical safety in patients with insulinoma (79). {Lys^40^ [Ahx-hydrazinonicotinamide (HYNIC)-^99m^Tc]NH2}exendin-4 was also developed as an alternative to [Lys^40^(Ahx-DTPA-^111^In)NH2]exendin-4 (80). Separately, Jodal et al. reported on the use of ^68^Ga-labeled exendin-4 with a chelator, 1-(1,3-carboxypropyl)-1,4,7-triazacyclononane-4,7-diacetic acid (NODAGA) (81). The conjugation of the chelator to resident lysines at position 12 or the C-terminally attached lysines at position 40 resulted in favorable binding and kinetics for the peptide, in accordance with their high specific uptake in GLP-1R-positive murine tissues. Interestingly, the authors also reported that {Lys^12^[(^68^Ga)NODAGA]}exendin-4 was more stable than {Lys^40^[(^68^Ga)NODAGA]}exendin-4 in human blood plasma. In this context, an exendin-4 derivative labeled with ^111^In *via* BnDTPA and Ahx attached to the epsilon amino group at the lysine-12 residue, [Lys^12^(^111^In-BnDTPA-Ahx)]exendin-4, has been synthesized (**Figure 4A**) (14). This probe has a high affinity for GLP-1R, and the introduction of In-BnDTPA at lysine 12 does not affect the affinity for GLP-1R. The radiochemical purity exceeded 95%, and specific activity at the end of synthesis was superior to that of the ^111^In-labeled exendin-3 derivative (12, 14). A preclinical biodistribution study of [Lys^12^(^111^In-BnDTPA-Ahx)]exendin-4 showed higher and more stable murine pancreatic uptake compared with [^111^In]BnDTPA-exendin (9-39) (14, 75). In addition to good pancreatic uptake with probe internalization, low probe uptake and rapid clearance in the surrounding organs (including the liver and kidneys) are desirable properties for *in vivo* β-cell imaging probes (14, 77). For [Lys^12^(^111^In-BnDTPA-Ahx)]exendin-4, the pancreas-to-liver uptake ratios increased in a time-dependent manner, and the pancreas-to-kidney uptake ratios remained stable, which enhanced the contrast and allowed for clear visualization of the murine pancreas in SPECT images (**Figure 4B**) (14).

As for PET imaging, ^68^Ga, ^18^F, ^64^Cu, and zirconium-89 (^89^Zr)-labeled exendin-4 derivatives have been developed (39). In particular, ^68^Ga-labeled exendin-4 derivatives have been well researched in preclinical and clinical studies, with the goal of overcoming the low spatial resolution and relatively high kidney uptake of SPECT probes by using ^68^Ga for PET imaging. In addition to NODAGA (81), several chelator moieties such as DOTA, 1,4,7-triazacyclononane-triacetic acid (NOTA), and desferrioxamine B (DFO) have been conjugated to exendin-4 at various positions. [Lys^40^(Ahx-DOTA-^68^Ga)NH2]exendin-4 showed significant uptake in insulinomas developed in RipTag2 mice and proved to be a potential alternative to [Lys^40^(Ahx-DTPA-^111^In)NH2]exendin-4 (80). Exendin-4 peptide radioiodinated at Tyr^40^ side by side with [Nle^14^, Lys^40^(Ahx-DOTA-^68^Ga)NH2]exendin-4 showed high binding to INS-1 cells and fast internalization kinetics, yielding *in vivo* tumor visualization in mice bearing INS-1 xenografts (82). A similar peptide, ^68^Ga-DO3A-*VS*-Cys^40^-exendin-4, also demonstrated GLP-1R-mediated accumulation in the pancreas of rats and cynomolgus monkeys (83). As for NOTA conjugation, an exendin-4 derivative comprising leucine at position 14 and NOTA-conjugated Met-Val-Lys (MVL) sequence with Cys^40^ ([^68^Ga]NOTA-MVK-Cys^40^-Leu^14^)exendin-4 showed comparable tumor uptake and reduced kidney uptake in INS-1 mouse xenografts to that of a control agent without the cleavable MVL sequence (84). Similarly, [Lys^40^(Ahx-DFO-^68^Ga)NH2]exendin-4 demonstrated high serum stability and high and specific *in vivo* accumulation in mice bearing RINm5f xenografts (85). Several clinical studies have been conducted using ^68^Ga-labeled exendin-4 derivatives, mainly for the purpose of detecting insulinoma, and have reported successful visualization of insulinomas and acceptable clinical safety (86–89). The potential benefits of radioisotopes with longer half-lives have been investigated using ^64^Cu (half-life, 12.8 h) and ^89^Zr (half-life, 78.4h). Although ^64^Cu-DO3A-*VS*-Cys^40^-exendin-4 showed strong accumulation in INS-1 xenografts and transplanted islets (90), [^64^Cu]NODAGA-exendin-4 failed to visualize the murine pancreas in *in vivo* PET imaging due to high kidney and liver uptake (91). The other chemical modifications with ^64^Cu labeling still have difficulties avoiding high kidney and liver uptake (92). As for ^89^Zr, [Lys^40^(Ahx-DFO-^89^Zr)NH2]exendin-4 showed comparable biological performance with [Lys^40^(Ahx-DFO-^68^Ga)NH2]exendin-4, whereas [Lys^40^(Ahx-DFO-^89^Zr)NH2]exendin-4 revealed long kidney retention times (85). In this context, fluorine-18 appears to be one of the most favorable radioisotopes for nuclear medicine imaging due to its positron emission energy and potential for high resolution and widespread use in current clinical settings (93, 94). However, preclinical and clinical studies of ^18^F-labeled exendin-4 derivatives are still relatively limited (94). Against expectations, most previous reports on ^8^F-labeled exendin-4 derivatives have demonstrated high non-specific uptake even in the kidneys (39, 89, 94). For example, [^18^F]FB-exendin-4 showed relatively high liver and kidney uptake, leading to low contrast in *in vivo* PET images (95). Several potential strategies for reducing non-specific uptake and kidney uptake have therefore been discussed, including the incorporation of highly lipophilic groups, albumin and albumin-binding domains, and nanoparticles (94, 96–98). Certain modifications such as [^18^F]fluoropentyl maleimide (FPenM)-[Cys^40^]exendin-4 and [^18^F]fluoronicotinamide (FNEM)-[Cys^40^]exendin-4 have improved renal clearance and tumor-to-kidney contrast in mice bearing INS-1 tumors (93, 99). Exendin-4 conjugated with polyethylene glycol might prove to be a promising alternative, given that PEGylation is a well-established technique for increasing the probe’s molecular weight and stability in circulation and for improving its specific uptake (100).

## Quantification of β-Cell Mass Using Glucagon-Like Peptide-1R-Targeted Imaging

Advances in GLP-1R-targeted imaging have opened the door to non-invasive BCM quantification in the whole intact pancreas, representing a breakthrough in uncovering the pathophysiology of diabetes mellitus and guiding the development of novel therapeutic strategies in diabetes. Non-invasive BCM quantification methods have been investigated based on the use of probes achieving successful visualization of pancreatic β cells. Consequently, these studies have been performed primarily as preclinical studies using small animals and SPECT imaging with ^111^In-labeled probes (11–13, 35, 101–103). Although SPECT appears to present drawbacks in *in vivo* quantification analysis due to the decay and diffusion of its low-energy gamma rays, recent studies have demonstrated that SPECT could yield sufficient quantitative information, especially in rodents (12, 13, 101, 102).

^111^In-labeled exendin-3 derivatives have been reported as useful for quantifying rat BCM with *ex vivo* SPECT scans of resected pancreata (12, 103). In Brown Norway rats with and without alloxan treatment, ^111^In-[Lys^40^]exendin-3 demonstrated a good correlation between pancreatic uptake (determined by the quantitative analysis of SPECT scans) and BCM (determined by the histological analysis of immunohistological staining with insulin antibodies) (r = 0.83) (12). Similarly, [Lys^40^([^111^In]DTPA)]exendin-3 showed a distinctively different uptake in resected pancreas between healthy and alloxan-treated diabetic Brown Norway rats. The *ex vivo* pancreatic uptake of [Lys^40^([^111^In]DTPA)]exendin-3 determined by SPECT showed linear correlations with histological BCM (r^2^ = 0.52) and with BCM based on optical projection tomography (r^2^ = 0.77) (104). Importantly, the probe’s pancreatic uptake was not correlated with alpha cell mass (105). Moreover, severe insulitis and hyperglycemia did not affect the linear correlation between the pancreatic probe uptake and histological BCM in nonobese diabetic (NOD) mice (106). Although mice were reported to show relatively higher exendin uptake in the exocrine pancreas compared with rats (66, 107), [Lys^12^(^111^In-BnDTPA-Ahx)]exendin-4 demonstrated good linear correlations between *ex vivo* pancreatic uptake and histological BCM in several diabetic model mouse strains such as NOD (r = 0.90) (102), db/db (35), and RCS-10 mice (r^2^ = 0.75) (11). These ^111^In-labeled exendin-3 and exendin-4 derivatives can therefore be promising probes for BCM quantification using *ex vivo* SPECT imaging.

However, *ex vivo* SPECT scans for BCM quantification can provide only limited opportunities for BCM observation during an individual’s lifetime and are invasive for obtaining pancreatic samples. Establishing an *in vivo* SPECT analysis method for BCM quantification is therefore essential for performing non-invasive BCM evaluations. Analyzing *in vivo* rodent SPECT images is more difficult than with *ex vivo* images due to two factors: indistinguishable pancreatic margins in CT images and the influence of renal probe uptake on the analysis of pancreatic regions of interest (101). To solve these issues, several attempts have been examined in addition to various chemical modifications on exendin skeletons to reduce renal uptake as described in the previous section Visualization of β cells: Glucagon-like peptide-1 receptor-targeted imaging. Mathijs et al. performed unilateral nephrectomy prior to SPECT scans to reduce the influence from the left kidney in rats (71). According to this protocol, [Lys^40^([^111^In]DTPA)]exendin-3 showed a good correlation between pancreatic uptake determined by *in vivo* SPECT/CT and histological BCM in biobreeding diabetes-prone rats with unilateral nephrectomy (r = 0.89) (108). Furthermore, dual probe injection methods have been examined to distinguish the pancreas from other abdominal organs. ^123^I-labeled L-phenylalanine and ^99m^Tc-demobesin-4 have been employed with ^111^In-labeled exendin-3, improving the correlation between *ex vivo* pancreatic uptake and that determined by *in vivo* SPECT image analysis (r = 0.83 and r = 0.92, respectively) (71, 109). However, these methods still required simultaneous nephrectomy, which might alter probe biodistribution, and could not adequately cover the volume of pancreatic regions of interest for BCM evaluation of the whole pancreas (71, 101, 109). A method for the *in vivo* SPECT imaging analysis of mice without the need for nephrectomy or a secondary probe has subsequently been developed using [Lys^12^(^111^In-BnDTPA-Ahx)]exendin-4 (101). The exclusion of the peripheral space 2.7 mm from the kidney surface on SPECT/CT images can remove the influence of renal uptake and can cover over 40% of the entire pancreatic volume, which provides a reliable estimate of the mean uptake value for the entire pancreas (101). According to this method, the correlation between *ex vivo* pancreatic uptake and that determined by *ex vivo* SPECT scans was almost perfect (r = 0.99). *In vivo* SPECT imaging analysis demonstrated good correlation between pancreatic uptake determined by *in vivo* SPECT scans and histological BCM in NOD (r = 0.89) (102) and db/db mice (r = 0.93 and 0.84) (35, 103). These correlations were higher or comparable to those achieved by methods that require nephrectomy and secondary probes.

As for clinical research, SPECT scans using [Lys^40^([^111^In]DTPA)]exendin-3 have been performed on patients with type 1 diabetes mellitus (12). In this study, the pancreatic uptake estimated with a SPECT imaging analysis showed an approximately 60% reduction in the patients with type 1 diabetes compared with the healthy participants. However, the pancreatic probe uptake between the two groups overlapped, and high interindividual variations were observed. Further clinical studies for non-invasive BCM evaluation are therefore warranted, including with other exendin-4 derivatives and PET imaging.

## Application of GLP-1R-Targeted Imaging in Diabetes Mellitus

The establishment of *in vivo* SPECT imaging analysis using [Lys^12^(^111^In-BnDTPA-Ahx)]exendin-4 has enabled the longitudinal observation of BCM changes in diabetic model mice with and without interventions. In NOD mice, a reduction in pancreatic probe uptake in *in vivo* SPECT images was longitudinally observed in the mice that developed hyperglycemia, whereas no significant changes in pancreatic probe uptake in the *in vivo* SPECT images were observed in the mice that did not develop hyperglycemia (102). In db/db mice, longitudinal *in vivo* SPECT observations revealed a spontaneous reduction in pancreatic probe uptake (35, 103) and that diet-restriction attenuated the reduction in BCM loss (35). *In vivo* SPECT observations also observed that canagliflozin (a sodium glucose transporter-2 inhibitor) and DS-8500a (a G protein-coupled receptor 119 agonist) attenuated the progression of BCM loss in db/db mice (34, 103). Although chronic hyperglycemia might affect probe uptake *via* changes in GLP-1R expression levels on the β-cell membrane surface (110), the pancreatic uptake of [Lys^12^(^111^In-BnDTPA-Ahx)]exendin-4 in SPECT images could replicate the BCM relationship among these model mice with different glycemic states (35, 102, 103). Moreover, the pancreatic uptake of [Lys^12^(^111^In-BnDTPA-Ahx)]exendin-4 maintained a linear correlation with histological BCM, even among diabetic and non-diabetic RCS-10 mice (11). The pancreatic uptake of the probe consists of combined probe intensities of cell-surface bindings to GLP-1R and intracellular accumulations. Consequently, it doesn’t simply reflect cell-surface GLP-1R expression level changes but also it can be largely affected by probe internalization and accumulation in pancreatic tissues, which may contribute to maintain a linear correlation with BCM. ^111^In-exendin-4 SPECT/CT is therefore useful for the non-invasive longitudinal investigation of BCM *in vivo* and can help reveal the BCM preservation effects of each intervention.

The monitoring of transplanted islet grafts is another potential application of GLP-1R-targeted imaging (111). In rodents with intramuscular islet transplantation, [Lys^40^([^111^In]DTPA)]exendin-3 showed non-invasive visualization of islet grafts and quantification of islet graft volume on SPECT (13, 110). Although exendin-3 probe uptake in islet grafts might be affected by the glycemic state *via* GLP-1R expression (presumably due to the small number of islets in a graft), the linear correlation between probe uptake and islet graft volume was maintained (110). In mice that underwent intraportal islet transplantation, ^68^Ga-DO3A-*VS*-Cys^40^-exendin-4 PET visualized human transplanted islets in the liver (112). As a clinical report of a patient with the autologous transplanted islets in the left brachioradialis muscle, [Lys^40^(Ahx-DTPA-^111^In)NH2]exendin-4 SPECT demonstrated focal accumulation in the left forearm at the site of islet transplantation (113). These results suggest the major potential of non-invasive monitoring of islet grafts; further investigations are expected for future clinical applications.

Lastly, GLP-1R-targeted imaging can be employed to better select antidiabetic drugs including GLP-1R agonists. The pancreatic uptake of [Lys^12^(^111^In-BnDTPA-Ahx)]exendin-4 is reflected in the *in vivo* glucose-lowering effects of dulaglutide in diabetic RCS-10 mice, which suggests that the pancreatic uptake value could predict responders and non-responders to dulaglutide therapy (11). Given that the probe’s pancreatic uptake was significantly correlated with BCM and GLP-1R mRNA expression, GLP-1R-targeted imaging can be a predictive indicator of the efficacy of not only GLP-1R agonists but also other antidiabetic drugs.

## Conclusions and Further Perspectives

The last two decades of research have achieved remarkable advances in non-invasive β-cell imaging and BCM evaluation, including the discovery of probe target molecules, the development of suitable radioisotope-labeled chemically modified probes, and the establishment of an analysis method for image-based signals using SPECT and PET. In particular, exendin-4 based derivatives for SPECT and PET appear to be promising candidates for non-invasive BCM evaluations. At least in rodents, [Lys^12^(^111^In-BnDTPA-Ahx)]exendin-4 SPECT is a useful tool for *in vivo* longitudinal BCM monitoring. Further clinical investigation is necessary to meet the medical needs of BCM evaluation in diabetes. Although various novel chemical modifications and structures of exendin peptides have yet to be investigated, further comparative studies including head-to-head comparisons among the candidate probes and techniques are warranted to standardize non-invasive BCM evaluation methods using GLP-1R-targeted imaging techniques.

## Author Contributions

TM performed a literature search and wrote and edited the manuscript. HF contributed to the plan and discussion. NI reviewed and edited the manuscript. All authors contributed to the article and approved the submitted version.

## Funding

This work was supported by grants from the Japan Foundation for Applied Enzymology (Front Runner of Future Diabetes Research) (to TM), MSD Life Science Foundation (to TM), and Manpei Suzuki Diabetes Foundation (to NI).

## Conflict of Interest

NI received clinical commissioned/joint research grants from Daiichi Sankyo, Terumo, and Drawbridge Inc.; speaker honoraria from Kowa, MSD, Astellas Pharma, Novo Nordisk Pharma, Ono Pharmaceutical, Nippon Boehringer Ingelheim, Takeda, Eli Lilly Japan, Sumitomo Dainippon Pharma, and Mitsubishi Tanabe Pharma; scholarship grants from Kissei Pharmaceutical, Sanofi, Daiichi Sankyo, Mitsubishi Tanabe Pharma, Takeda, Japan Tobacco, Kyowa Kirin, Sumitomo Dainippon Pharma, Astellas Pharma, MSD, Eli Lilly Japan, Ono Pharmaceutical, Sanwa Kagaku Kenkyusho, Nippon Boehringer Ingelheim, Novo Nordisk Pharma, Novartis Pharma, Teijin Pharma, and Life Scan Japan.

The remaining authors declare that the research was conducted in the absence of any commercial or financial relationships that could be construed as a potential conflict of interest.
